# Infusion of Silver–Polydopamine Particles into Polyethersulfone Matrix to Improve the Membrane’s Dye Desalination Performance and Antibacterial Property

**DOI:** 10.3390/membranes11030216

**Published:** 2021-03-19

**Authors:** Hazel Lynn C. Maganto, Micah Belle Marie Yap Ang, Gian Vincent C. Dizon, Alvin R. Caparanga, Ruth R. Aquino, Shu-Hsien Huang, Hui-An Tsai, Kueir-Rarn Lee

**Affiliations:** 1 School of Chemical, Biological and Materials Engineering and Sciences, Mapúa University, Manila 1002, Philippines; maganto1213@gmail.com (H.L.C.M.); arcaparanga@mapua.edu.ph (A.R.C.); 2 School of Graduate Studies, Mapúa University, Manila 1002, Philippines; 3 R&D Center for Membrane Technology and Department of Chemical Engineering, Chung Yuan Christian University, Taoyuan 32023, Taiwan; gian.dizon23@yahoo.com (G.V.C.D.); huian@cycu.edu.tw (H.-A.T.); 4 General Education Department, Colegio de Muntinlupa, Mayor J. Posadas Avenue, Sucat, Muntinlupa City 1770, Metro Manila, Philippines; ruthraquino@yahoo.com; 5 Department of Chemical and Materials Engineering, National Ilan University, Yilan 26047, Taiwan; 6 Research Center for Circular Economy, Chung Yuan Christian University, Taoyuan 32023, Taiwan

**Keywords:** silver, polydopamine, polyethersulfone, nanofiltration, mixed-matrix membrane

## Abstract

The advancement in membrane science and technology, particularly in nanofiltration applications, involves the blending of functional nanocomposites into the membranes to improve the membrane property. In this study, Ag-polydopamine (Ag-PDA) particles were synthesized through in situ PDA-mediated reduction of AgNO_3_ to silver. Infusing Ag-PDA particles into polyethersulfone (PES) matrix affects the membrane property and performance. X-ray photoelectron spectroscopy (XPS) analyses confirmed the presence of Ag-PDA particles on the membrane surface. Field emission scanning electron microscopy (FESEM) and atomic force microscopy (AFM) describe the morphology of the membranes. At an optimum concentration of Ag-PDA particles (0.3 wt % based on the concentration of PES), the modified membrane exhibited high water flux 13.33 L∙m^−2^∙h^−1^ at 4 bar with high rejection for various dyes of >99%. The PES_Ag-PDA0.3_ membrane had a pure water flux more than 5.4 times higher than that of a pristine membrane. Furthermore, in bacterial attachment using *Escherichia coli*, the modified membrane displayed less bacterial attachment compared with the pristine membrane. Therefore, immobilizing Ag-PDA particles into the PES matrix enhanced the membrane performance and antibacterial property.

## 1. Introduction

One of the major challenges facing the application of membrane-based processes in dye desalination is the gradual accumulation and proliferation of bacteria on the membrane that causes the formation of biofilm [1,2,3]. Biofilms are formed when microorganisms attached to and grow on the membrane surface [4,5,6,7,8]. Subsequently, the formation of biofilms on the membrane surface decreases the membrane permeability and life span, which could lead to an increase in operational cost [4,9,10]. To prevent the formation of biofilm, developing an antibacterial surface to reduce the magnitude of the initial bacterial attachment has been the main focus of numerous research efforts [1]. In the dyeing process, large amounts of inorganic salts are used as an electrolyte for the migration, adsorption, and fixation of the dyestuff to the cellulose material [11,12]. This results in the production of a significant quantity of wastewater with combined salt and dye that must be treated and separated using effective methods [13].

Polyethersulfone (PES) is regarded as one of the engineering polymers for nanofiltration (NF). This polymer is soluble in a wide range of solvents and has good film-forming properties [14]. Although PES is considered an important polymeric material, the hydrophobic nature of PES restricts its membrane applications [15]. Modification of the PES surface to enhance its surface hydrophilicity is one of the solutions. An example is by introducing hydrophilic moieties such as sulfone, carboxyl (–COOH), hydroxyl (–OH), and amine (–NH_2_) [4]. There are different methods to perform this solution: surface grafting [16], ultraviolet (UV) [17,18], plasma treatment [19], and the blending method [20,21,22,23,24,25]. Another method is the blending of hydrophilic materials without affecting the thermal and mechanical properties of the PES backbone [4]. Moreover, a blending technique could tailor the membrane physicochemical property to enhance the membrane performance [4,26].

In water treatment processes, the attachment of bacteria, viruses, and other microorganisms in the PES surface are inevitable because of their presence in nearly all aquatic environments. The physicochemical properties of a membrane such as the surface and cross-sectional morphologies, surface roughness, hydrophilicity, and surface charge are the main factors that determine the initial bacterial cell adhesion [2]. These properties are considered as a crucial influence for subsequent biofilm development on the membrane surface. Recent studies are focused on exploring new methods specifically in using nanoparticles to enhance membrane efficiency and antibacterial property [27].

Nanoparticles embedded to a polymeric membrane resulting in a mixed-matrix membrane have been proven to modify the polymeric structures affecting the conveyance of molecules through membrane pores, which improves the performance [28]. Mixed-matrix membranes have many advantages: outstanding selectivity, low-cost manufacturing, high packing density of polymeric materials, high mechanical strength, and good regeneration capability [28]. Nanomaterials, specifically the metallic materials, are used for their safety, heat stability, and long-lasting activity [29]. Nanomaterials such as copper [30], silver [31], gold [32], and titanium [33] exhibit promising physicochemical properties resulting in significant levels of antibacterial activity [34,35]. However, incorporating these materials on different substrates remains a challenge [35]. Among the metallic nanomaterials, silver exhibited high toxicity toward many types of bacteria, but low toxicity for humans [36,37]. Thus, using silver particles to incorporate the antibacterial function becomes an important solution to membrane challenges on biofilm formation. Yet, silver incompatibility to polymers resulted in leaching problems [29,38,39,40].

One of the effective solutions to this problem is to fabricate a hybrid nanomaterial. These hybrid materials could consist of an inorganic–organic nanoparticle. A mussel-inspired material, polydopamine (PDA), has been reported to provide good adhesion in most of the substrate. PDA has been applied as a polymer coating or modifier of nanoparticles for membranes in many membrane applications [41,42,43,44,45,46,47]. After introducing PDA, the stability and membrane property were improved. In addition, PDA could reduce the silver precursor in situ to silver nanoparticles to its surface and become a hybrid nanoparticle [35,48,49,50,51,52]. The metal ion bonding is related to functional groups found in PDA including amino, catechol, carboxy, o-quinone, imine, and phenol groups [35,48,53,54]. Therefore, this study focuses only on the application of PDA and Ag-PDA nanoparticles into the fabrication of the PES mixed-matrix membrane for dye separation. Ag-PDA particles were synthesized through an in situ reduction of AgNO_3_ in PDA particles. Afterwards, Ag-PDA particles were embedded in the PES matrix to improve the membrane separation efficiency for dye desalination with better antibacterial property. This could be a promising solution to impart an antibacterial material into a nanofiltration membrane with high separation efficiency [55].

## 2. Materials and Methods

### 2.1. Materials

PES powder (VERADEL 3000MP with M_w_ = 64,000 g mol^−1^–68,000 g mol^−1^) was supplied by Solvay Company, Tokyo, Japan. N,N-Dimethylacetamide (DMAc, 99.5%), as a solvent of PES, was acquired from the Tedia Company Inc., USA. AgNO_3_ (purity: ≥99.80%) was purchased from Alfa Aesar, Heysham, Lacashire, England. Ammonium hydroxide (28%) was bought from Nihon Shiyaku Industries Ltd., Tokyo, Japan. Ethanol (purity: 99.5%) was produced from Echo Chemical Co. Ltd., Taiwan. Dopamine hydrochloride (purity: ≤100%) was obtained from Sigma-Aldrich, Steinheim, Germany. Methylene Blue (purity: ≥96.0%; high purity grade), Procion Blue H-5R (purity: <100%), Rose Bengal (purity: <100%), and Direct Red 80 (purity: <100%) were manufactured by Alfa Aesar, Heysham, Lacashire, England. Orange G (purity: >85%) was a product of Acros Organics, Belgium. Direct Red 23 (purity: 30%) was procured from Sigma-Aldrich, Saint Louis, MO, USA. Brilliant Blue R (purity: <100%) was delivered by Tokyo Chemical Industry Co., Ltd., Tokyo, Japan. Sodium chloride (NaCl, 99.0%), sodium sulfate (Na_2_SO_4_, 99.0%), magnesium chloride (MgCl_2_, 98.0%), and magnesium sulfate (MgSO_4,_ 99.5%) were provided by Sigma-Aldrich, Saint Louis, MO, USA. *Escherichia coli* was distributed by Food Industry Research and Development Institute Bioresource Collection and Research Center, Hsinchu, Taiwan.

### 2.2. Preparation of Ag-PDA Nanoparticles

PDA particles were prepared through an alkaline water–ethanol environment through the self-polymerization of a dopamine monomer. The alkaline water–ethanol mixture was prepared by using deionized water (90 mL), ethanol (40 mL), and aqueous ammonia (3 mL). This mixture was stirred at 60 rpm and 30 °C for 30 min. Subsequently, dopamine hydrochloride (0.5 g) was dissolved in deionized water (10 mL) to guarantee dispersion of the dopamine monomer. The solution was transferred directly to the alkaline water–ethanol mixture (133 mL) that was kept stirred for 30 h at 60 rpm and 30 °C using a magnetic stirrer (Cimarec+™ Stirrer Series). The volume ratio of water to ethanol mixture is 2.575:1. Afterwards, the PDA particles were rinsed through centrifugation using deionized water five times. The dark brown particles were freeze-dried and stored in a vacuum ball at room temperature for further use.

**Figure 1:**
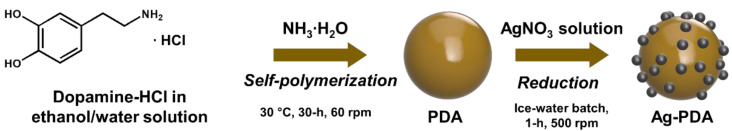
Synthesis of polydopamine (PDA) and Ag-PDA particles. PDA particles were centrifuged and washed before the functionalization of Ag on its surface.

Ag-PDA nanoparticles were prepared with a similar method from Wu and coworkers [49]. Figure 1 presents the synthesis of Ag-PDA particles. Silver nitrate (0.8354 g) was added to deionized water (290 mL) and was stirred in an ice-water bath at 500 rpm. The aqueous ammonia was dropped (0.35 µL) slowly into the solution until the solution became transparent. Then, 0.1 g of PDA particles were dispersed in deionized water (10 mL). Afterwards, the PDA solution was transferred into the freshly prepared [Ag(NH_3_)_2_]^+^ ion aqueous solution. The solution was kept stirred at 500 rpm in an ice-water bath for 1 h to ensure a complete reaction. The Ag-PDA nanocomposite particles were washed five times using deionized water through centrifugation. Lastly, the Ag-PDA nanocomposite particles were dried in a freeze dryer and then stored in a vacuum ball at room temperature until used.

### 2.3. Preparation of Mixed-Matrix Membranes

The PES mixed-matrix membranes were prepared through a nonsolvent induced phase separation method. Table 1 lists the composition and viscosity of the PES solutions. The PES and modified PES membranes were fabricated using a casting solution of 19 wt % PES, 81 wt % DMAc, and 0.1–0.3 wt % particles (added based on the amount of PES). To avoid aggregation of the particles in the solution, the particles (PDA or Ag-PDA) were dispersed in DMAc using ultra-sonication for 1 h. After dispersing the particles, PES powder was dissolved in the solution and was stirred at 250 rpm and 50 °C. Then, the solution was degassed for 1 h at a temperature of 30 °C. Subsequently, the resulted solution was cast on a glass plate using a 100 μm casting knife at a relative humidity of 50%–80% and at room temperature. The glass plates were promptly submerged into deionized water (30 °C) for phase separation. Finally, the prepared membranes were stored in deionized water for at least 24-h prior to testing.

### 2.4. Characterization of Ag-PDA Nanoparticles and Membranes

Transmission electron microscopy (TEM, JEOL JEM-2100, Tokyo, Japan) was used to examine the morphology of the particles. Field emission scanning electron microscopy (FESEM, S-4800, Hitachi Co., Tokyo, Japan) was utilized to capture the images of the particles for the measurement of particle size. The crystallinity of the particles was determined using X-ray diffraction (XRD, Model D8 Advance Eco, Bruker, Billerica, MA, USA) equipped with a crystallographic database with a 2θ angle recorded in the range of 10°–80°. The surface and cross-section morphologies of the PES and PES-modified membranes were examined using field emission scanning electron microscopy (FESEM, S-4800, Hitachi Co., Tokyo, Japan). The surface topography of the PES and PES-modified membranes were characterized by atomic force microscopy (AFM, Bruker, Billerica, MA, USA) with images obtained using NanoScope Analysis software. The functional group composition of the PES and PES-modified membranes was evaluated by an attenuated total reflectance-Fourier-transform infrared spectroscopy (ATR-FTIR, Perkin Elmer Spectrum 100 FTIR, Waltham, MA, USA). The elemental composition of the PES and PES-modified membranes was surveyed using X-ray photoelectron spectroscopy (XPS, ThermoFisher Scientific Inc., Waltham, MA, USA). The surface hydrophilicity of the PES and PES-modified membranes was quantified at room temperature using a water contact angle (PD-VP Model, Kyowa Interface Science Co. Ltd., Niiza-City, Saitama, Japan). The surface charge of the membranes was measured using a zeta potential measurement, which was carried out by using a dynamic light scattering instrument (Zeta Nano ZS, Malvern, Cambridge, UK).

### 2.5. Evaluation of Membrane Performance

Prior to the nanofiltration test, membranes were washed using deionized water. Membranes were installed in a lab-scale crossflow filtration setup, which is similar to our previous work [56]. Before measuring the pure water flux, membranes were pre-pressurized at 4.5 bar for 1 h to ensure a steady-state condition. After 1 h, the pressure was adjusted to 4 bar with a retentate flowrate of 0.75 L∙min^−1^ at 30 °C to measure the pure water flux. Pure water flux (*J*) and solute rejections (*R*) were calculated using the following equations:

(1) $$J=\frac{m}{\rho \times A\times t}$$

(2) $$R\left(\%\right)=\left(1-\frac{C_{p}}{C_{f}}\right)\times 100\%$$

where *m* (kg) is the mass of the permeate, *t* (h) is the time of permeate collection, *A* (cm^2^) is the effective membrane surface area of 12.57 cm^2^, *ρ* (1 kg∙L^−1^) is the water density, and the solute concentrations of permeate and feed solutions are *C_p_* and *C_f_*, respectively. The feed and permeate concentrations for salt were measured using a SevenMulti Mettler Toledo AG (Mettler Toledo Group, Columbus, OH, USA). The absorbance of the different dyes was obtained using the UV-visible spectrophotometer (PowerWave XS, Biotek, Winooski, VT, USA). All the membranes were pre-pressurized for 1 h at 4.5 bar using pure water as feed to ensure the steady-state stability.

### 2.6. Bacterial Attachment Test

The bacterial attachment tests were conducted using *E. coli* as the model bacteria. The bacteria were modified with a green fluorescent protein (GFP), following the method used by Hsiao et al. in 2014 [57]. Membrane samples having a diameter of 1 cm were placed and washed in a well plate with phosphate buffer solutions (PBS). Then, 1 mL of each bacterial solution was added into each well until the samples were fully immersed. The samples were incubated with 1 mL of bacterial culture added at a temperature of 37 °C, where the fresh bacterial solution was replaced with the same concentration every 6 h for 24 h. After the incubation period, the bacterial solution was removed, and each sample was washed thrice with PBS to remove any unattached bacteria from the membrane surface. Afterwards, the samples were observed at a magnification of 200× to examine the bacterial attachment on the membrane using confocal laser scanning microscopy (NIKON CLSM A1R, Melville, NY, USA). Photographs of bacterial attachment were taken from 2 various sites on each sample, and each condition was repeated 3 times. To determine the average cell density attached to the membrane surface, open-source software ImageJ was used.

## 3. Results and Discussion

### 3.1. Characterization of Particle

**Figure 2:**
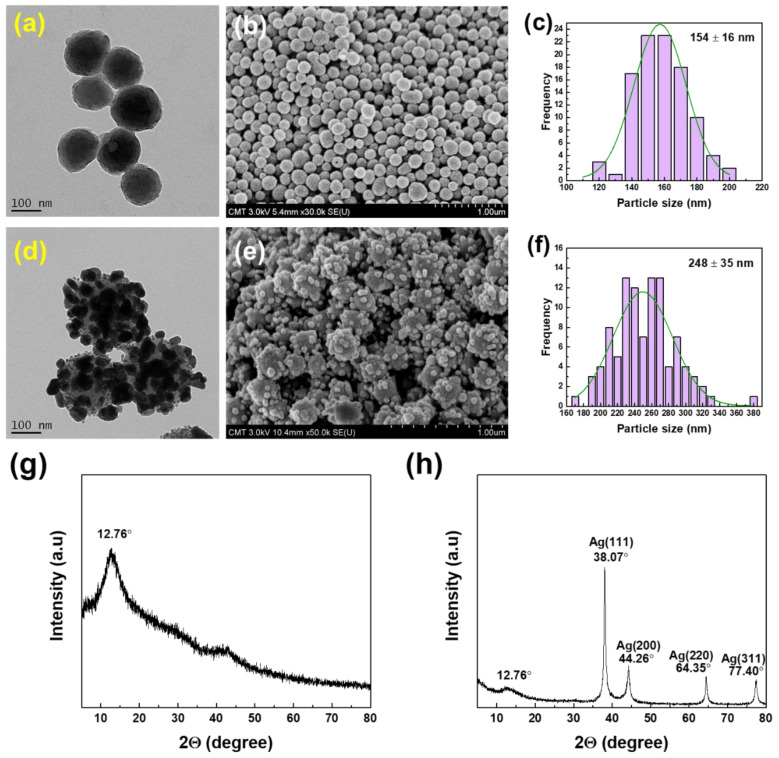
TEM and FESEM images, size distribution, and XRD spectra of (**a**–**c**,**g**) PDA and (**d**–**f**,**h**) Ag-PDA particles.

Figure 2 shows the TEM, SEM, size distribution, and XRD patterns of PDA particles and Ag-PDA particles. PDA particles had an average diameter of 154 ± 16 nm, whereas the Ag-PDA particles had an average size of 248 ± 35 nm. The metal particles are believed to adhere to the O-site and N-site of PDA that eventually acted as the foundation for the initial formation of metal nanoparticles through the atom-by-atom growth in a continuous reduction of metal ions [35]. The catechol groups of PDA were able to discharge electrons when oxidized into the corresponding quinone group and initiate reduction processes of metallic cations [58]. According to Yang et al. [59], electrons released by the oxidation of catechol to quinone can reduce silver ions in the solution phase. At the same time, the O- and N-based ligand sites in PDA could serve as anchors for the resulting Ag nanoparticles. Metal nanocomposites were successfully synthesized through the functional groups present in PDA as a versatile feature of PDA [60].

Figure 2g,h confirm the functionalization of Ag in the PDA surface. In the XRD pattern, the broad peak at 12.767° was ascribed to the amorphous structure of the PDA particles [61]. In addition, the XRD patterns of Ag-PDA nanocomposite particles demonstrated the existence of PDA loaded with silver showing five XRD diffraction peaks at 38.1°, 44.3°, 64.4°, 77.4°, and 81.6° which corresponded to the diffraction peaks of (111), (200), (220), (311), and (222) lattice planes. These diffraction peaks indicate a face centered-cube (FCC) phase of the Ag crystal. In addition, the intensity of the peak (111) was far stronger than that of the other peaks, which indicated the rapid growth of Ag crystal on (111). In the early stage of Ag nanoparticles formation, the nucleation of the Ag^0^ phase by the chemical potential stabilization of the Ag^+^ ions at the catechol sites is expected to occur with limited numbers. As the reaction time increases, a large amount of catechol groups are fully utilized for the reaction with Ag^+^ ions, and the growing process between the generated Ag^0^ atoms becomes dominant rather than generating newly nucleated Ag nanoparticles [62]. This signifies that the reduction process did not alter the properties and crystallography structure of PDA.

### 3.2. Characterization of the Membranes

**Figure 3:**
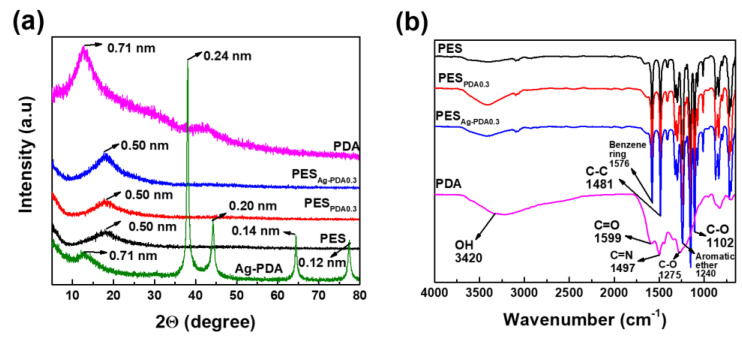
(**a**) XRD spectra of PES; PES_PDA0.3_; PES_Ag-PDA0.3_; PDA; and Ag-PDA. (**b**) Attenuated total reflectance-Fourier-transform infrared spectroscopy (ATR-FTIR) spectra of polyethersulfone (PES) and modified membranes. The samples were tested at room temperature.

Figure 3 indicates the ATR-FTIR and XRD spectra of PES, PES_PDA0.3_, and PES_Ag-PDA0.3_ membranes. Figure 3a illustrates the XRD patterns of particles (PDA and Ag-PDA) and XRD diffraction patterns of the fabricated membranes (PES, PES_PDA0.3_, and PES_Ag-PDA0.3_). The characteristic peaks of PDA particles and Ag-PDA nanocomposite particles were not observed in the XRD analyses. This suggests that the particles were uniformly distributed in the fabricated membrane because of the low concentration of particles to the casting solution.

Figure 3b indicates the major peaks of PES at 1576, 1481, 1240, and 1102 cm^−1^, which were attributed to benzene ring, C–C bonds, aromatic ether, and C–O bonds of PES structure. Although many functional groups such as carboxy, amino, imine, hydroxyl, and phenol groups are present in PDA [35], the absorption vibration at 3420 cm^−1^ was considered to be the most distinguished peak of the PDA particles obtained. This was attributed to the O–H stretching vibrations indicating the presence of the hydroxyl group. PDA also have peaks at 1599, 1497, and 1275 cm^−1^, which are ascribed to C=O, C=N, and C–O of the PDA structure. After modification, the strong characteristic peaks observed at 3420 cm^−1^ exhibited noticeable changes of PES_PDA0.3_ and PES_Ag-PDA0.3_ compared to pristine PES, indicating the successful incorporation of the particles to the membrane. Furthermore, the elemental chemical composition of the PES and PES-modified membranes was analyzed by XPS. Table 2 summarized the elemental composition of the membranes. The presence of N and Ag on the surface for the modified membranes confirmed that PDA or Ag-PDA was embedded in the membrane.

**Figure 4:**
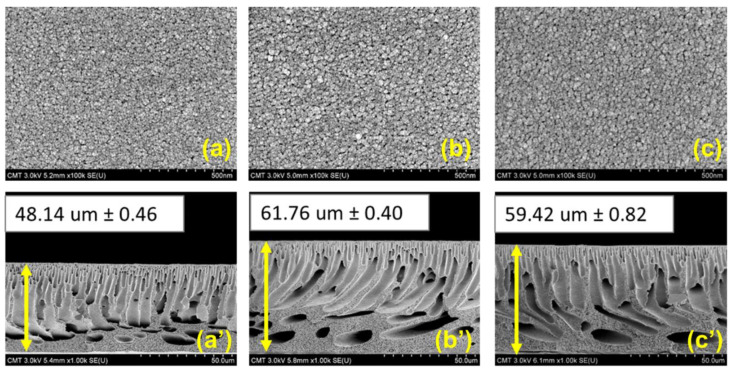
FESEM images of (**a**,**a**’) PES; (**b**,**b**’) PES_PDA0.3_; (**c**,**c**’) PES_Ag-PDA0.3_. Surface images magnification = ×100k; cross-sectional images magnification = ×1k.

**Figure 5:**
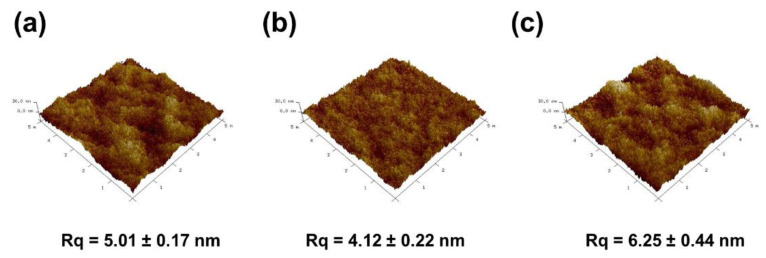
Three-dimensional atomic force microscopy (AFM) images of (**a**) PES; (**b**) PES_PDA0.3_; (**c**) PES_Ag-PDA0.3_. Lateral scale = 5 µm; vertical scale = −30 to 30 nm.

Figure 4 and Figure 5 demonstrate membrane morphologies of the PES and modified PES membranes. The membrane surface of pristine PES exhibited no noticeable pores and a relatively smooth surface. Furthermore, there was no observable significant difference between the surface pore morphology of the PES and that of the modified membranes (PES_PDA0.3_ and PES_Ag-PDA0.3_). This type of membrane surface is obtained when liquid–liquid demixing occurs instantaneously, resulting in a membrane with a dense top layer [1,14]. In order to further explore the influence of the PDA particles and Ag-PDA nanocomposite particles on the morphology of PES membranes, the cross-sectional SEM images of membranes are shown in Figure 4a’–c’. All membranes exhibited an asymmetric structure with a dense thin top layer, tightly packed finger-like morphology at the top sublayer, and macrovoids at the bottom support layer. Blending the nanoparticles brought a change to the membrane by affecting the kinetics and thermodynamics of the system [63]. The presence of hydrophilic materials in the casting solution increased the mass exchange rate between the solvent and non-solvent which, results in bigger channels [63]. Furthermore, after the incorporation of PDA and Ag-PDA particles containing 0.3 wt % concentration, the membrane thickness increases from 48.14 ± 0.46 µm to 61.76 ± 0.40 µm and 48.14 ± 0.46 µm to 59.42 ± 0.82 µm, respectively. Adding hydrophilic nanoparticles into the polymer solution promotes thermodynamic instability, resulting in an increase in membrane thickness [64]. In addition, PES_PDA0.3_ and PES_Ag-PDA0.3_ had similar thickness. During the phase inversion process, the hydrophilic nanofillers enhanced the demixing rate, thus resulting in a thicker membrane [56].

Figure 5a reveals that gaps between peaks and valleys on the PES were uniform and smooth, indicating the low surface roughness of the investigated membranes. With the addition of PDA particles to the PES, a smoother surface (Figure 5b) was obtained from 5.01 ± 0.17 nm to 4.12 ± 0.22 nm. On the other hand, the membrane topography (Figure 5c) increased from 5.01 ± 0.17 nm to 6.25 ± 0.44 nm with the addition of Ag-PDA particles. The root-mean-square surface roughness (Rrms) values of the two modified membranes compared to the corresponding pristine showed contrary results. The values of the modified membranes revealed the successful incorporation of particles to different membranes.

**Figure 6:**
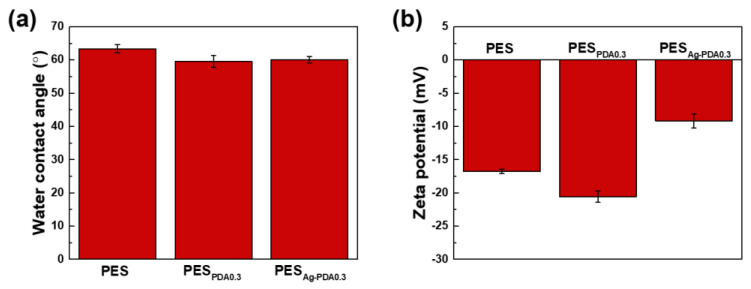
(**a**) Water contact angle and (**b**) zeta potential of the membranes. The water contact angle was measured after 1 min contact of the droplet on the membrane surface. Zeta potential was measured at pH = 7.

The hydrophilicity of PES membrane is measured using the time-dependent dynamic water contact angle (WCA) test (Figure 6a). The PES membrane exhibited a water contact angle of 63.38 ± 1.28°. After modification through the addition of PDA and Ag-PDA particles, decreases of water contact angle measurements to 59.51 ± 1.77° and 60.08 ± 0.96° were obtained for PES_PDA0.3_ and PES_Ag-PDA0.3_, respectively. The small changes in water contact angle were caused by the small amount of nanoparticles on the membrane surface. PDA is a hydrophilic nanoparticle because of its abundance in catechol, quinone, and amine groups [48]. An increase in the membrane surface hydrophilicity could facilitate the water solubilization and diffusion through the membrane and therefore enhance water permeation [65]. The introduction of hydrophilic functional groups on the surface without modifying the backbone of the polymer membrane is regarded as an attractive methodology for the modification of PES. As shown in Figure 6b, the charge density of the membrane surface plays a very important role in the separation performance of the membranes. The nanofiltration membrane often carries a negatively charged surface. A more negatively charged membrane was obtained with the addition of PDA particles to the membrane, while a less negative membrane was obtained with the addition of Ag-PDA particles. The negatively charged surface of the membrane was caused by sulfonic and/or carboxylic acid groups on a skin membrane layer [6]. The zeta potential illustrated that a lesser negative charge membrane was obtained with Ag-PDA nanoparticles compared to PDA particles. Ag-PDA particles could neutralize some of the negative moieties in the PES matrix, thus giving less negatively charged surface.

### 3.3. Performance Evaluation

**Figure 7:**
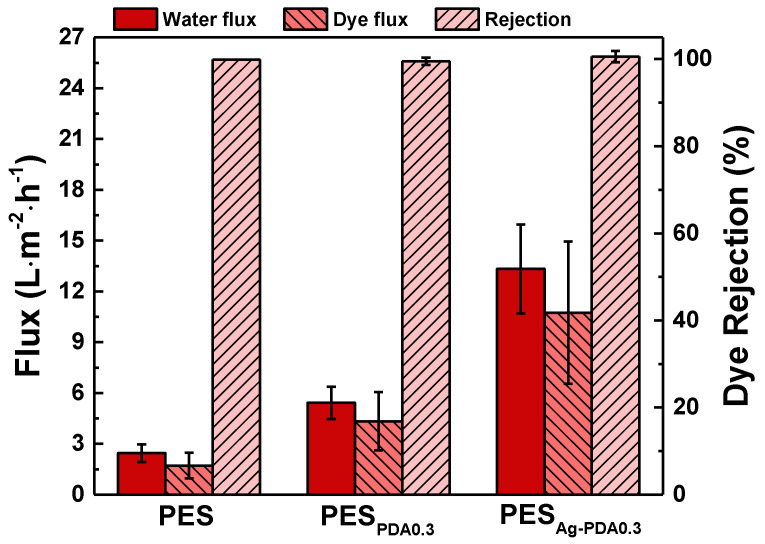
Performance comparison of PES, PES_PDA0.3_, and PES_Ag-PDA0.3_. Feed = 50 ppm Rose Bengal; Operating pressure 4 bar; Operating temperature = 30 °C.

Figure 7 describes the membrane performance of PES, PES_PDA__0.3_, and PES_Ag-PDA0.3_ membranes in terms of permeation flux and dye rejection using 50 ppm Rose Bengal (operating conditions of 4 bar, 30 °C, and 0.75 L∙min^−1^). The pristine PES membrane had a pure water flux of 2.45 ± 0.5 L∙m^−2^∙h^−1^, whereas PES_PDA0.3_ and PES_Ag-PDA0.3_ had a higher flux of 5.42 ± 0.95 and 13.33 ± 2.62 L∙m^−2^∙h^−1^, respectively. The contact angle, cross-sectional morphologies, surface topography, and surface charge were also affected by the addition of the particles. The incorporation of Ag-PDA particles to the membrane enhanced the permeation fluxes, which were attributed to the functional groups embedded in the membrane. PDA, embedded into the membrane, initiated the nucleation of PES during the phase separation. Infusing hydrophilic materials into the polymer matrix would cause an easier phase inversion with water. The hydrophilic material added could also serve as nucleation sites for the formation of the membrane. This resulted in the detachment of polymer chains from the surface of the nanoparticles and the formation of interface void channels between the polymer and fillers across the skin layer [22]. Since Ag-PDA particle morphology had a bigger particle size, its capacity to develop new pathways for the water molecules to pass through is higher. A decrease in the surface negativity charge shown in zeta potential also suggests that this could be the reason for the increase in permeation fluxes. Furthermore, the interaction of water with the surface of membrane has increased the transport of water molecules [22]. Furthermore, the increased permeation flux of the PES_Ag-PDA_ was also because of the rougher surface of PES_Ag-PDA_. All the membranes exhibited a dye rejection using Rose Bengal of ≈99.9%. The rejection was maintained because the amount of particles was enough to prevent the aggregation of the particles in the polymer matrix, which can cause defects. Therefore, embedding Ag-PDA particles enhanced the membrane separation efficiency.

**Figure 8:**
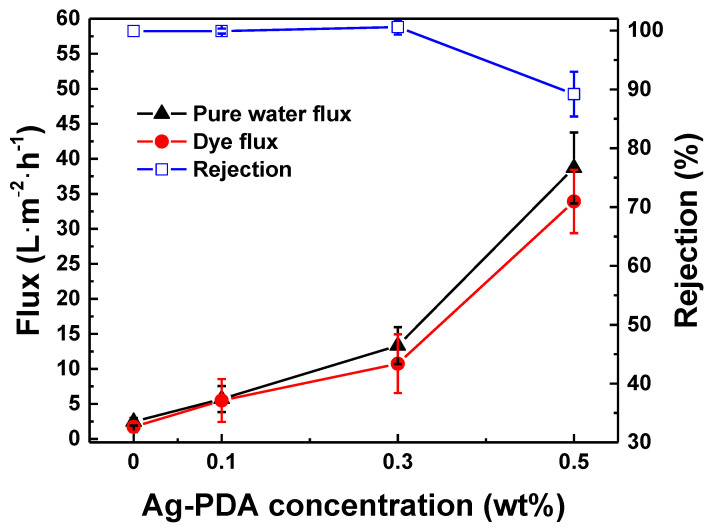
Effect of Ag-PDA concentration on membrane performance. Feed = 50 ppm. Rose Bengal; Operating pressure 4 bar; Operating temperature = 30 °C.

Figure 8 plots the effect of Ag-PDA concentration on membrane performance. Increasing the concentration from 0 to 0.3 wt %, the rejection remains the same (>99%). In addition, there was an increase in pure water flux from 2.45 ± 0.5 to 13.33 ± 2.62 L∙m^−2^∙h^−1^. However, the dye rejection decreased to 89.20 ± 3.81% when concentration was increased to 0.5 wt % Ag-PDA. This decrease in the dye rejection was because of the high content of the Ag-PDA particles. At a high content, some of the Ag-PDA particles could leach out during the phase inversion process, resulting in more pores on the membrane matrix. Furthermore, it was also possible that particles aggregate on the polymer matrix, which could lead to defects. Thus, the optimal concentration of Ag-PDA particles in the PES matrix was 0.3 wt %.

**Figure 9:**
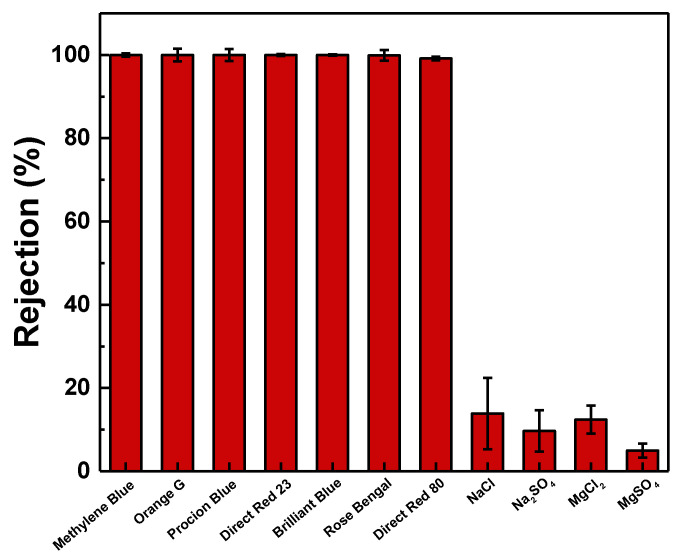
PES_Ag-PDA0.3_ performance on different dye and salt solution. Feed = 50 ppm of Dye; Operating pressure 4 bar; Operating temperature = 30 °C.

Seven types of water-soluble dyes (Methylene Blue, Orange G, Procion Blue H-5R, Direct Red 23, Brilliant Blue, Rose Bengal, and Direct Red 80) with different molecular weights (Table 3) were used as feed solution with a concentration of 50 ppm to investigate the rejection capacity of the PES_Ag-PDA0.3_ membrane (Figure 9). The membrane exhibited high rejections for Methylene Blue (99.9 ± 0.39%), Orange G (99.9 ± 1.51%), Procion Blue H-5R (99.9 ± 1.47%), Direct Red 23 (99.9 ± 0.24%), Brilliant Blue (99.9 ± 0.14%), Rose Bengal (99.90 ± 1.28%), and Direct Red 80 (99.13 ± 0.46%), demonstrating its potential in dye recovery from textile wastewater. In addition, the negative surface charge of the PES_Ag-PDA0.3_ membrane (−9.15 ± 1.05 mV) is beneficial for high dye rejection due to the Donnan exclusion effects [66,67]. Therefore, anionic dye molecules were prohibited to pass through the membrane channels, causing a higher rejection for anionic dyes. The size exclusion effect only had a limited influence on the dye rejection, since Methylene Blue with a lower molecular weight of 319.86 g.mol^−1^ resulted in 99.9 ± 0.39% rejection. However, water-soluble dyes tend to form clusters due to the hydrophobic interactions between the aromatic rings of adjacent dye molecules and/or the inter-molecular hydrogen bonding [22,66,68]. Therefore, a larger size of dye in an aqueous solution was obtained than its corresponding monomeric dye molecule, resulting in high retention for small dye molecules [27]. Four types of inorganic salt (NaCl, Na_2_SO_4_, MgCl_2_, and MgSO_4_) were used to investigate the salt rejection of PES_Ag-PDA0.3_ membrane. PES_Ag-PDA0.3_ membrane had low salt rejections: MgSO_4_ (4.97 ± 1.66%); Na_2_SO_4_ (9.68 ± 4.93%); MgCl_2_ (12.40 ± 3.36%); NaCl (13.86 ± 8.54%). Therefore, PES_Ag-PDA0.3_ had high separation efficiency for dyes.

Table 4 lists the membrane performance of this work and compared it with other reported literature. Only a PES-based membrane was considered for the comparison. Several particles had been used in the literature that improved the membrane performance and antifouling property. Comparing the membrane in our study to that of the literature, there is a trade-off between the membrane performance. This means that permeability is high with a high dye rejection for the large molecular weight of dyes; otherwise, permeability is low for the membrane that could reject low molecular weight dyes.

### 3.4. Bacterial Attachment Test

**Figure 10:**
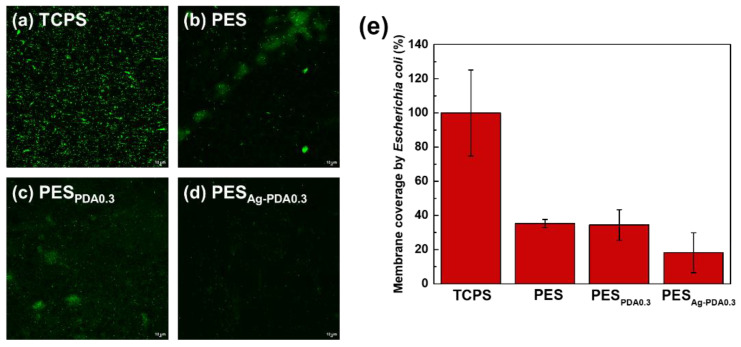
Antibacterial capability of the membranes assessed via the attachment of *E. coli.* (**a**–**d**) Representative confocal micrographs of the membranes, and (**e**) quantified bacterial coverage via ImageJ software.

Figure 10 represents the bacterial attachment test for *Escherichia coli* to the membrane in comparison with the attachment of bacteria from the control (TCPS), PES, and PES_Ag-PDA0.3_ membranes. The results showed that the PES_Ag-PDA0.3_ membrane exhibits good antibacterial ability against *Escherichia coli* with bacterial attachment decreasing to 11%. This is due to the antibacterial capacity of Ag nanoparticles decorated on the PDA particle surfaces, which is incorporated into the PES membrane. Incorporating the Ag nanoparticles into the membranes resulted in a rougher surface that directly increases the bacterial adhesion with a tendency to defend the bacterial cells from fluid shear forces. The first one is the disruption of fluid flow. Rough surfaces create areas of low shear where the forces that might remove attached bacteria are significantly reduced [6]. The Ag particles are believed to inhibit the bacterial cells in contact with the membrane surfaces by using PDA as the template to immobilize its function in the membrane. When the cell membrane of the microorganism comes in contact with silver, silver ion damages the cell wall. Furthermore, the formation of reactive oxygen species (ROS) causes damage to the cell due to oxidative stress. This suggested that ROS could be produced at the cell membrane that may lead to irreversible damage to DNA replication, affecting cell division and metabolic processes [34].

## 4. Conclusions

The mixed-matrixed PES membranes were fabricated with PDA or Ag-PDA nanoparticles through phase inversion method. The hydrophilic property of PDA or Ag-PDA nanoparticles enhanced the demixing rate of the PES solution, resulting in more porous membrane. Compared with PES and PES_PDA0.3_, PES_Ag-PDA0.3_ delivered high permeation flux (5.4 times higher than pristine membrane) with high dye rejections (Methylene Blue = 99.9 ± 0.39%, Orange G = 99.9 ± 1.51%, Procion Blue H-5R = 99.9 ± 1.47%, Direct Red 23 = 99.9 ± 0.24%, Brilliant Blue = 99.9 ± 0.14%, Rose Bengal = 99.90 ± 1.28%, and Direct Red 80 = 99.13 ± 0.46%). The optimal concentration for Ag-PDA in the PES matrix was 0.3 wt %. Furthermore, in bacterial attachment test using *Escherichia coli,* PES_Ag-PDA0.3_ exhibited good antibacterial property. Therefore, embedding Ag-PDA particles improved the membrane property and performance.

## Figures and Tables

**Table 1:** Composition and viscosity of polymer solution.

Solution	PES (wt %)	DMAc (wt %)	Particle (wt %) ^a^	Viscosity (cp)
PES	19	81	0	424.47 ± 2.10
PES_PDA0.3_	19	81	0.3	438.93 ± 0.49
PES_Ag-PDA0.1_	19	81	0.1	456.60 ± 1.56
PES_Ag-PDA0.3_	19	81	0.3	462.73 ± 0.51
PES_Ag-PDA0.5_	19	81	0.5	502.47 ± 1.50

^a^ Added based on the amount of PES.

**Table 2:** Surface elemental composition of the membranes from XPS analysis.

Membrane	C (%)	O (%)	S (%)	N (%)	Ag (%)
PES	77.70 ± 1.30	15.43 ± 0.95	6.88 ± 0.37	-	-
PES_PDA0.3_	74.35 ± 0.42	18.32 ± 0.28	6.21 ± 0.24	1.12 ± 0.13	-
PES_Ag-PDA0.3_	69.98 ± 1.12	22.01 ± 1.44	5.39 ± 0.28	2.40 ± 0.28	0.22 ± 0.27

**Table 3:** Molecular weights of the dye.

Different Dyes Dye Concentration: 50 ppm	Molecular Weights (g∙mol^−1^)
Methylene Blue	319.85
Orange G	452.38
Procion Blue	637.44
Direct Red 23	813.73
Brilliant Blue	826.98
Rose Bengal	1017.65
Direct Red 80	1373.08

**Table 4:** Performance comparison of PES mixed matrix membrane of this study and other literature.

Membrane	Operating Pressure (bar)	Permeability (L∙m^−2^∙h^−1^∙bar^−1^)	Dye Concentration (ppm)	Molecular Weight of Dye (g/mol)	Dye Rejection (%)	NaCl Concentration (ppm)	NaCl Rejection (%)	Reference
PES	4.00	0.61	50.00	973.67	99.00	-	-	This study
PES_PDA0.3_	4.00	1.35	50.00	973.67	99.51	1000.00	-	This study
PES_Ag-PDA0.3_	4.00	3.33	50.00	319.85	99.99	1000.00	12.40	This study
PES + HNTs-SO3H	4.00	18.25	1000.00	576.49	80.00	1000.00	3.00	[69]
PES + CNs	5.00	18.00	984.20	813.73	98.00	2000.00	8.00	[70]
PES + AFNPs	4.00	4.19	30.00	637.55	98.00	200.00	18.00	[71]
PES + G-TA	4.00	8.00	50.00	637.55	92.61	-	-	[72]
PES-CDs	3.00	25.5	100.00	983.5	98.9	200.00	20.1	[49]
PES + rGO/TiO_2_	5.00	9.00	100.00	1079.60	85.00	-	-	[73]
PES + TETA-MWCNT	10.00	8.30	50.00	407.98	98.00	1000.00	23.00	[74]
PES + ZnO/MWCNTs	4.00	4.18	30.00	637.55	95.00	-	-	[20]
PES + BNNS	4.00	8.00	10.00	973.67	88.00	-	-	[75]
PES + MoS_2_-PSBMA	6.00	18.10	400.00	991.8	98.2	1000.00	1.1	[76]

HNTs-SO_3_H: sulfonated halloysite nanotubes; CNs: cellulose nanosheets; AFNPs: adipate ferroxane nanoparticles; G-TA: goethite–tannic acid; CDs: carbon quantum dots; rGO: reduced graphene oxide; TETA-MWCNT: triethylenetetramine functionalized multiwall carbon nanotube; A-HNTs: 3-aminopropyltriethoxysilane functionalized halloysite nanotubes; ZnO/MWCNTs: zinc oxide-coated multiwalled carbon nanotubes; BNNS: boron nitride nanosheets; PSBMA: poly(sulfobetaine methacrylate).
